# Hydatid Brain Cyst in a Limping Child

**DOI:** 10.4269/ajtmh.22-0374

**Published:** 2022-11-14

**Authors:** Siham Elamour, Shalom Ben-Shimol, Israel Melamed

**Affiliations:** ^1^ Pediatric Infectious Disease Unit, Soroka University Medical Center, Beer Sheva, Israel;; ^2^ Faculty of Health Sciences, Ben Gurion University of the Negev, Beer Sheva, Israel;; ^3^ Neurosurgery Unit, Soroka University Medical Center, Beer Sheva, Israel

A 6-year-old Bedouin boy presented with a limp and headache. On physical examination, bradycardia, left facial nerve palsy, and left hemiparesis were noted. Laboratory findings were notable for eosinophilia (eosinophil count of 890/μL). Brain computed tomography and brain magnetic resonance imaging revealed a brain cyst (6 cm in diameter) (Figure 1A and B). Abdominal ultrasound and chest radiography did not reveal additional cysts and blood serology (ELISA) for echinococcosis^1^^,^^2^ was negative. The brain cyst was fully removed surgically (Media 1).

**Figure 1.**
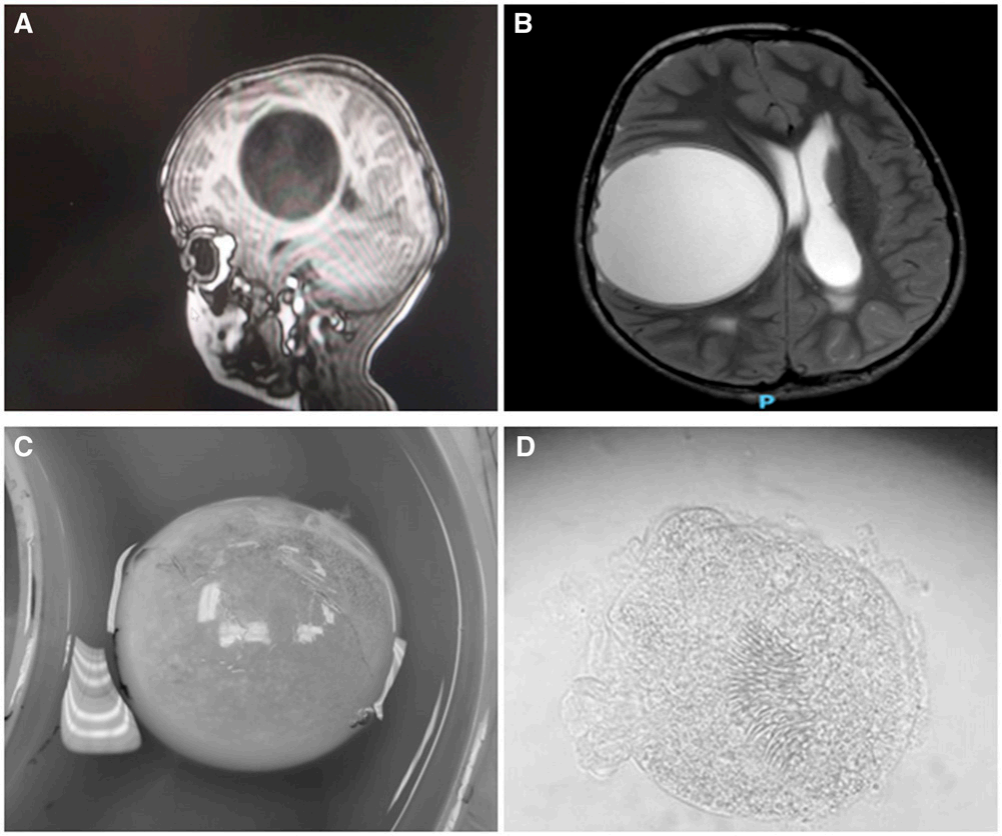
(**A**, **B**) Brain MRI revealed a 6 cm diameter brain cyst: (**A**) T1 sagittal view without contrast; (**B**) T2 axial view with contrast. (**C**) The brain cyst after removal. (**D**) Histopathologic examination of the cyst content after centrifugation 4 minutes 500 rpm viewed under microscope 4 hpf showing echinococcal larvae.

Histopathological examination of the brain cyst fluid showed several echinococcal larvae (Figure 1C and D), confirming cystic echinococcosis (CE) diagnosis. Other than larvae, no features of hydatid cyst were observed. Over the following 4 months, he was treated with albendazole (10 mg/kg/day) without complications. Sequential brain imaging showed considerable improvement (Figure 2), and the child had no complaints or remarkable findings on physical examination; there were no residual neurologic deficits on follow-up examination.

**Figure 2.**
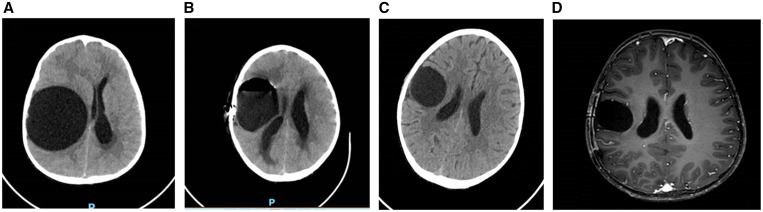
(**A**–**C**) Brain CT – axial view without contrast: (**A**) brain cyst with daughter cyst – pre surgery; (**B**) day 1, post-surgery; (**C**) 2 months post-surgery. (**D**) Brain MRI – axial T2 view without contrast, 4 months post-surgery.

*Echinococcus granulosus* is a tapeworm that causes zoonotic infection (echinococcosis or hydatid disease) and is transferred to humans, the intermediate hosts of the worm, by ingestion of contaminated food with the parasite’s eggs. Echinococcosis is endemic in southern Israel, especially in the Bedouin population, likely because of close daily contact between humans and animals, particularly stray dogs, the definitive host of *E. granulosus*. Humans develop cystic (larval) disease, mainly of the liver or the lungs, over long periods. Brain CE is relatively rare, reported only in 1% to 2% of all CE cases. Although CE in adults reflects transmission occurring many years earlier, the disease in children and animal hosts is indicative of recent transmission. Diagnosis requires a high index of suspicion, typical radiographic findings, and specific serology test, which has low sensitivity in extra-hepatic cases.^3^^,^^4^

## Supplemental files


Supplemental materials
